# Meetings of Societies

**Published:** 1928

**Authors:** 


### Meetings of Societies.

### Bristol Medico-Chirurgical Society.

*February 8th*, 1928.

Dr. W. K. Wills, President, in the Chair.

Dr. S. E. Dore read a paper on "**Radiation Therapy in Dermatology,**" reported on page 85 of our last issue.

---

*March 14th*, 1928.

Dr. W. K. Wills, President, in the Chair.

Dr. A. T. Todd gave a further account of his "**Chemo-therapeutic Researches on Cancer,**"[1] an appreciation of which appears on page 217.

This was preceded by a short address on the "**Colloidal State of Matter,**" by Professor Francis. The report of this address, which was illustrated by many experiments, appears on page 177.

In the discussion which followed Dr. Todd's paper, Dr. R. S. Statham spoke of fourteen cases of malignant disease of the pelvic organs treated with lead. In two instances he had observed great improvement. In some of those that had not been so fortunate cicatrisation following arrest of the growth had brought about ureteral obstruction. The relief from pain had been remarkable.

Dr. D. A. Alexander described the regression of a breast tumour under the influence of treatment. The patient had ultimately died of metastases.

Professor D. C. Rayner corroborated Dr. Statham's account of the favourable reaction of an ovarian carcinoma.

Mr. C. Ferrier Walters spoke of the help afforded by lead in the treatment of two malignant growths of the prostate, surgically removed.

---

*April 11th*, 1928.

Dr. A. L. Flemming in the Chair.

Mr. A. G. Timbrell Fisher, M.C., read a paper on "**Manipulative Surgery,**" part of which is reported on page 165.

[1] Bristol: J. W. Arrowsmith Ltd. 2s. 6d.

*May 9th*, 1928.

DR. W. K. WILLS, President, in the Chair.

DR. G. B. BUSH showed a group of radiograms illustrating bone pathology.

MR. G. W. ROSS showed a case of abdominal swelling, possibly hydatid disease. MR. C. A. MOORE mentioned that he had recently had a case of hydatid disease from Wales and asked what was proposed. The PRESIDENT suggested X-ray treatment on the assumption that this was a case of Hodgkin's disease.

MR. E. WATSON-WILLIAMS showed four cases of cancer of the tonsil in elderly men treated by selenium, with and without radium in addition. He emphasised the difficulties which arose from patients presenting themselves for treatment only when the disease was advanced, with enlargement of glands.

DR. P. WATSON-WILLIAMS discussed this point and the technique of treatment in inoperable cases.

DR. HERBERT ROGERS read a paper on "**The Estimation of Chlorides in the Cerebro-spinal Fluid,**" reported on page 197

---

### The Bath and Bristol Branch of the British Medical Association.

*May 30th*, 1928.

AT a Clinical Meeting, held on Wednesday, May 30th, at the University, MR. A. W. ADAMS showed a series of cases of club-foot, and cases of malignant disease of the alimentary tract.

DR. R. C. CLARKE showed a series of cases and pathological specimens of congenital heart disease.

MR. H. CHITTY showed cases of cervical rib and of congenital deformity.

MR. E. W. HEY GROVES showed cases illustrating the results obtained by operation in the treatment of injuries to limbs.

MR. C. A. MOORE showed a case of sciatic tumour.

DR. PERCY PHILLIPS showed cases of syringomyelia.

DR. A. T. TODD showed cases of amyotrophic lateral sclerosis and others.

MR. E. WATSON-WILLIAMS showed cases of atresia and hyperostosis of the external ear and others illustrating results obtained by operative treatment of malignant disease of the larynx.

DR. KENNETH WILLS showed cases of rodent ulcer and other dermatological diseases.

DR. A. D. FRASER showed pathological specimens.

